# Recapitulating Parkinson's disease pathology in a three-dimensional human neural cell culture model

**DOI:** 10.1242/dmm.038042

**Published:** 2019-04-09

**Authors:** Teresa R. Taylor-Whiteley, Christine L. Le Maitre, James A. Duce, Caroline F. Dalton, David P. Smith

**Affiliations:** 1 Biomedical Sciences Research Centre, Department of Bioscience and Chemistry, Sheffield Hallam University, Sheffield, South Yorkshire S1 1WB, UK; 2 School of Biomedical Sciences, The Faculty of Biological Sciences, University of Leeds, Leeds, West Yorkshire LS2 9JT, UK; 3 The ALBORADA Drug Discovery Institute, University of Cambridge, Cambridge Biomedical Campus, Hills Road, Cambridge CB2 0AH, UK

**Keywords:** 3D cell culture, Amyloid, Lewy body, Oligomer, Parkinson's, Synuclein

## Abstract

Extensive loss of dopaminergic neurons and aggregation of the protein α-synuclein into ubiquitin-positive Lewy bodies represents a major neuropathological hallmark of Parkinson's disease (PD). At present, the generation of large nuclear-associated Lewy bodies from endogenous wild-type α-synuclein, translationally regulated under its own promoter in human cell culture models, requires costly and time-consuming protocols. Here, we demonstrate that fully differentiated human SH-SY5Y neuroblastoma cells grown in three-dimensional cell culture develop Lewy-body-like pathology upon exposure to exogenous α-synuclein species. In contrast to most cell- and rodent-based PD models, which exhibit multiple diffuse α-synuclein aggregates throughout the cytoplasm, a single large nuclear inclusion that is immunopositive for α-synuclein and ubiquitin is rapidly obtained in our model. This was achieved without the need for overexpression of α-synuclein or genetic modification of the cell line. However, phosphorylation of α-synuclein within these inclusions was not observed. The system described here provides an ideal tool to screen compounds to therapeutically intervene in Lewy body formation, and to investigate the mechanisms involved in disease progression in synucleinopathies.

## Abstract (precis)

**Editor's choice:** This study describes a novel 3D cell culture model that recapitulates key features of neuropathology in synucleinopathies, including large, nuclear-associated α-synuclein and ubiquitin-positive inclusions.

## INTRODUCTION

The presence of large cell-associated protein aggregates is the key pathological hallmark commonly associated with many neurodegenerative disorders (Poewe et al., 2017). In Parkinson's disease (PD) and other synucleinopathies, the intrinsically disordered protein α-synuclein (α-syn) undergoes misfolding into amyloid fibrils (Poewe et al., 2017). These fibrils form the major protein component of intracellular deposits associated with Lewy bodies (LBs) in the cell soma, and Lewy neurites (LNs) in axons of surviving neurons (Theillet et al., 2016; Spillantini et al., 1997; Langston et al., 2015). The association of α-syn with pre-synaptic terminals from *in vitro* and *in vivo* models suggests a normal physiological role in the regulation of neurotransmitter release and synaptic function, but its role in disease remains poorly understood (Iwai et al., 1995; Kahle et al., 2000; Murphy et al., 2000; Nemani et al., 2010). Familial early-onset forms of PD are associated with mutations in the *SNCA* gene, encoding α-syn (Polymeropoulos et al., 1997; Singleton et al., 2003)*.* Genomic duplications, triplications and missense mutations (e.g. A53T, A30P, E46K and H50Q) all implicate α-syn in the pathogenesis of PD (Spatola and Wider, 2014). However, only 10% of cases are linked to a genetic basis of the disease, with the majority of cases having an unknown aetiology (Mcculloch et al., 2008; Wirdefeldt et al., 2011). Insights from *in vitro* and *in vivo* models suggest that α-syn acts as a ‘prion-like’ protein, with a propensity to misfold and form aggregates that promote cell-to-cell propagation, which assists in the spread of pathology (Braak et al., 2003; Li et al., 2008; Kordower et al., 2008; Danzer et al., 2009; Aulić et al., 2014; Hawkes et al., 2007).

The mechanisms underlying LB formation and the influence of α-syn pathology on disease pathogenesis remain poorly understood, largely due to the lack of whole-animal or cell-based models that recapitulate the development of these inclusions. One of the significant barriers in PD research surrounds the difficulty in obtaining cultures of the A9-subtype dopaminergic neuronal population that are specifically affected in the disease (Arenas et al., 2015). Several cell culture models have been used for studying PD, and to investigate the role of α-syn aggregation. These models include: non-patient-specific human cell lines (SH-SY5Y, HEK293, LUHMES); animal-derived cell lines (rat PC12, mouse N2a cells); stem cells, including induced pluripotent cell lines (iPSCs) and human mesenchymal (MSCs)/embryonic stem cells (ESCs); and primary animal-derived midbrain neuron cultures (Falkenburger et al., 2016; Smidt and Burbach, 2007). Each of these cell types has its own strengths and limitations; for example, the use of iPSCs that differentiate into dopaminergic neurons *in vitro* overcomes the ethical issues associated with using ESCs. However, culturing these cells is expensive and labour-intensive (as long as 75 days in culture), meaning that their use is inevitably out of reach for many research groups (Smirnova et al., 2016; D'Antonio et al., 2017). The cost incurred, time constraints and ethical framework required for animal-based research are again inhibitory for many laboratories.

To address the experimental and ethical issues of ESCs and animal models, alternative systems have been developed to model the complex pathogenesis of the disorder. Relatively few studies have observed the development of LB pathology without overexpressing high levels of human versions of α-syn (Volpicelli-Daley et al., 2011; Falkenburger et al., 2016). In addition, a predominant number of studies rely on the introduction of a familial mutation into α-syn (e.g. A53T) to increase aggregation propensity (Li et al., 2001; Koprich et al., 2017). Recombinant expression of wild-type (WT) human α-syn in *Drosophila* mirrors the formation of LB-like structures and neuronal loss, but this has unfortunately not been replicated in higher-order organisms or human-cell-based models (Feany and Bender, 2000). Interestingly, rodent models of PD that overexpress human α-syn by mutations in the *SNCA* gene (e.g. the M83 strain, overexpressing mutant human A53T α-syn) do develop inclusions but the anatomical distribution is widely variable among animals and often coincides with areas of substantial neuroinflammation (Sacino et al., 2014; Lee et al., 2002; Dawson et al., 2011; Fares et al., 2016). Importantly, impaired human α-syn fibrillisation can occur in rodent models due to an interaction with endogenously expressed mouse α-syn. Such interactions highlight a fundamental experimental caveat when investigating LB formation in mouse models or rodent-derived primary neuronal cultures (Fares et al., 2016). Species differences between rodent models (including primary rodent cell culture systems) make it difficult to model and extrapolate findings to human subjects (Goldie et al., 2014; Ioannidis, 2012; Pound and Bracken, 2014).

In previous human-cell-based models, limitations have also arisen from the use of traditional two-dimensional (2D) monolayers. Intracellular α-syn aggregates are often observed as multiple cytoplasmic punctate inclusions rather than recapitulating the typical large singular inclusions associated with human pathology (Danzer et al., 2009; Desplats et al., 2009; Aulić et al., 2014). Formation of LB-like pathology in human cell culture models has only been achieved through overexpression of α-syn, either driven from viral promoters or in cell lines not associated with the disease, such as those from human embryonic kidney lines (HEK-293) (Volpicelli-Daley et al., 2011; Luk et al., 2009; Sacino et al., 2013). Such viral promotors do not allow the study of transcriptional and translational modulations brought about during the disease state. To address these issues, *in vitro* 3D culture systems are increasingly being utilised for a multitude of disease models, with the aim of more closely mimicking an *in vivo* environment with tissue-like cell density and cell-to-cell as well as cell-to-extracellular-matrix contacts (Griffith and Swartz, 2006; Tang-Schomer et al., 2014; Choi et al., 2014; Smirnova et al., 2016). Taken together, the limitations of both *in vitro* and *in vivo* models demonstrate a greater need to develop a human-relevant culture system to model LB pathology.

The use of human-derived neuroblastoma cell lines, such as the SH-SY5Y catecholaminergic neuroblastoma cell line, remains a popular choice in PD research (Xicoy et al., 2017). Although the SH-SY5Y cell line does display genetic anomalies, it is important to note that nearly all pathways dysregulated in PD remain intact in this line (Krishna et al., 2014) and punctate intracellular inclusions can be induced by exposure to preformed amyloid material (Illes-Toth et al., 2015). In addition, upon differentiation, this proliferative immature neuroblast expresses the characteristic proteins associated with dopaminergic neurons, including tyrosine hydroxylase (TH) and dopamine transporter (DAT), making it a comparable model of the cell type affected in the disease state (Presgraves et al., 2004; Lopes et al., 2010; Kovalevich and Langford, 2013).

Differentiation to a fully mature neuronal phenotype can be achieved using all-trans retinoic acid (RA), followed by treatment with brain-derived neurotrophic factor (BDNF) (Encinas et al., 2000). This procedure yields a preferentially homogenous population of cells compared to other differentiation protocols, including short- or long-term treatment with RA alone (Encinas et al., 2000). Although the importance of using differentiated SH-SY5Y in PD research is becoming increasingly apparent, a large number of publications still either use the undifferentiated line, do not report their differentiation protocol or employ a differentiation protocol that solely utilises RA. These procedures then do not fully provide a mature neuronal phenotype (Goldie et al., 2014; Xicoy et al., 2017).

Here, we developed an easily replicable cell culture methodology that recapitulates the formation of LB-like inclusions. The Encinas et al. (2000) protocol was adapted to generate neuron-like cells in a 2D cell culture format that could subsequently be maintained within a 3D matrix. By incorporating the RA+BDNF-differentiated cells into a 3D matrix, we have been able to induce the formation of nuclear-associated ubiquitinated LB-like α-syn aggregates upon treatment with preformed α-syn oligomers. The formation of these LB-like aggregates was achieved without the need for overexpression of α-syn or modification of the genomic material.

## RESULTS

### Differentiated SH-SY5Y in 2D culture express markers of dopaminergic neurons

**Fig. 1.**
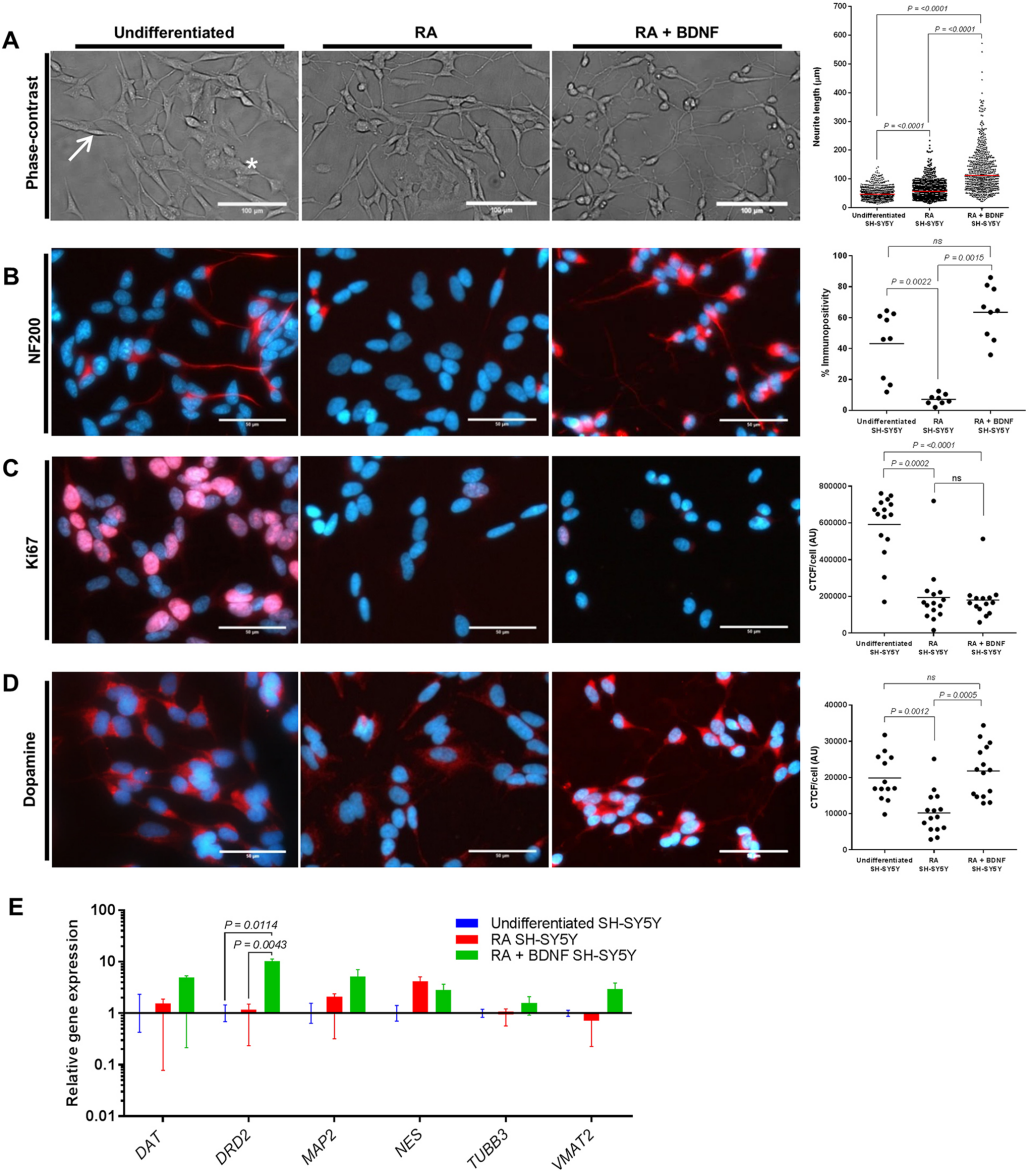
**Characterisation of SH-SY5Y cells following differentiation with RA alone or sequential treatment with** **RA+BDNF.** (A) Representative phase-contrast images of SH-SY5Y differentiated with RA alone and sequential treatment with RA+BDNF. Neurite length measurements were determined by measuring 200 neurites per treatment group, with three independent biological repeats. All data points are presented, with the mean shown as a straight line. Neuroblastic (‘N-type’; arrow) and substrate adherent (‘S-type’; asterisk) cells are shown. (B-D) Immunocytochemistry analysis of differentiated cultures stained for NF200, Ki67 and dopamine (DA) (red). Nuclei were visualised using DAPI staining (blue). Individual data points presented with mean (straight line) for % immunopositivity (B) and CTCF/cell data (C,D) of three independent biological repeats. (E) mRNA levels of genes specifically associated with dopaminergic neurons were evaluated by qPCR. Undifferentiated cells (blue), RA treatment group (red) and RA+BDNF treatment group (green). Relative gene expression normalised to reference genes (*ACTB* and *YWHAZ*) and undifferentiated SH-SY5Y. Neuronal (*TUBB3* and *MAP2*) and dopaminergic (*DAT*, *DRD2* and *VMAT2*) markers showed increased expression in the RA+BDNF group (green) relative to the undifferentiated cells (blue). Immature neuronal marker (*NES*) expression was not significantly changed following differentiation with RA or RA+BDNF. Data presented as mean±s.e.m. of at least three independent experiments. (A-E) Differences between treatments were tested for significance using the Kruskal–Wallis test with *post hoc* Dwass-Steel-Chritchlow-Fligner for >6 data points and Conover-Inman for <6 data points. *P-*values are displayed in the figures. Scale bars: 100 µm (A); 50 µm (B-D).

Controversy remains as to whether RA or RA+BDNF promotes SH-SY5Y cell differentiation and the development of a terminal neural dopaminergic phenotype (Korecka et al., 2013; Xicoy et al., 2017; Lopes et al., 2010). Thus, transcriptional and translational analysis of key neuronal markers was carried out to limit assumptions regarding the phenotypic state of the SH-SY5Y cell line used here. Undifferentiated SH-SY5Y cells display a characteristic morphology of two distinct phenotypes: neuroblastic (‘N-type’; Fig. 1A, arrow); and substrate adherent (‘S-type’; Fig. 1A, asterisk). In agreement with others (Korecka et al., 2013; Forster et al., 2015; Ito et al., 2017), upon treatment with RA or RA+BDNF, the neuroblastic SH-SY5Y cells differentiated to a more neuron-like phenotype. Average neurite lengths and standard deviations of 51.22±22.87 µm (undifferentiated), 64.91±33.07 µm (RA) and 130.50±77.91 µm (RA+BDNF) were observed (Fig. 1A), with interquartile ranges of 34.43-62.53, 40.83-80.85 and 74.83-164.8 µm, respectively. Significantly longer neurite length was observed in both RA and RA+BDNF groups compared to undifferentiated cells, with increased network complexity in the RA+BDNF group (Fig. 1A). Following treatment with RA, the non-differentiating ‘S-type’ population was increasingly visible at day 5 and, if continuously cultured up to 14 days, under these conditions, progressively overgrew the cultures (data not shown). The addition of BDNF and withdrawal of RA promoted the development of a homogenous population of cells with a negligible amount of ‘S-type’ cells surviving (Encinas et al., 2000).

Key to the development of an effective cell culture model of PD is the expression of relevant neuronal markers. Cells at all stages of differentiation were stained with antibodies for the neuron markers neurofilament 200 (NF200) (Fig. 1B) and βIII*-*tubulin (encoded by *TUBB3*) (data not shown). NF200 staining was reduced following differentiation with RA (7±3%) compared to undifferentiated SH-SY5Y (43±21%), potentially relating to an increased population of ‘S-type’ cells within the culture. NF200 immunopositivity was increased in RA+BDNF (64±17%) compared to undifferentiated and RA groups. Ki67 is a protein associated with cell proliferation that is present in the G_1_, S and G_2_ phases of cell cycling and mitosis but absent in the G_0_ resting state. Undifferentiated neuroblastoma cultures expressed this marker [591,943±171,477 corrected total cell fluorescence (CTCF)/cell]. A significant reduction in expression seen in the RA (193,629±160,908 CTCF/cell) and RA+BDNF (180,465±105,445 CTCF/cell) cells indicate that they are terminally differentiated (Fig. 1C). Tyrosine hydroxylase (TH) is a key enzyme in the production and homeostasis of dopamine (DA) within catecholamine neuronal cell types (Daubner et al., 2011) and is expressed in all three cell types (Fig. S1). An anti-DA antibody that has been demonstrated to be specific for DA through cross-reactivity studies (Banks et al., 2017) was used to determine the presence of this neurotransmitter. These terminally differentiated SH-SY5Y cells were also shown to retain a dopaminergic phenotype, whereby DA immunoreactivity was significantly increased in RA+BDNF (21,800±7126 CTCF/cell)-treated cultures compared to undifferentiated cells (19,884±6371 CTCF/cell) or those treated with RA (10,216±5823 CTCF/cell) (Fig. 1D).

Evaluation of the mRNA levels after each differentiation protocol by quantitative real-time PCR (qPCR) was used to determine the transcriptional levels of genes specifically associated with dopaminergic neurons (Fig. 1E). Increased expression of the neuronal markers βIII-tubulin (*TUBB3*) and microtubule-associated protein 2 (*MAP2*) upon RA+BDNF treatment confirmed the development of a more neuronal phenotype than observed with RA-treated or undifferentiated SH-SY5Y cells. Nestin (*NES*), an immature neuronal marker, was not significantly changed by differentiation with either RA alone or RA+BDNF treatment. A greater dopaminergic phenotype in RA-BDNF differentiated cultures was observed, indicated by elevated expression levels of DAT, DA receptor D2 and vesicular monoamine transporter member 2 when compared to the other two cell populations (Fig. 1E). Overall, these observations are in line with previously published work on differentiated SH-SY5Y cell lines in which RA+BDNF treatment was utilised, and demonstrate that these conditions result in terminally differentiated cells expressing many of the key markers of a dopaminergic neuron (Ito et al., 2017; Agholme et al., 2010; Xicoy et al., 2017).

### Exogenous α-syn oligomers induce intracellular aggregation

**Fig. 2.**
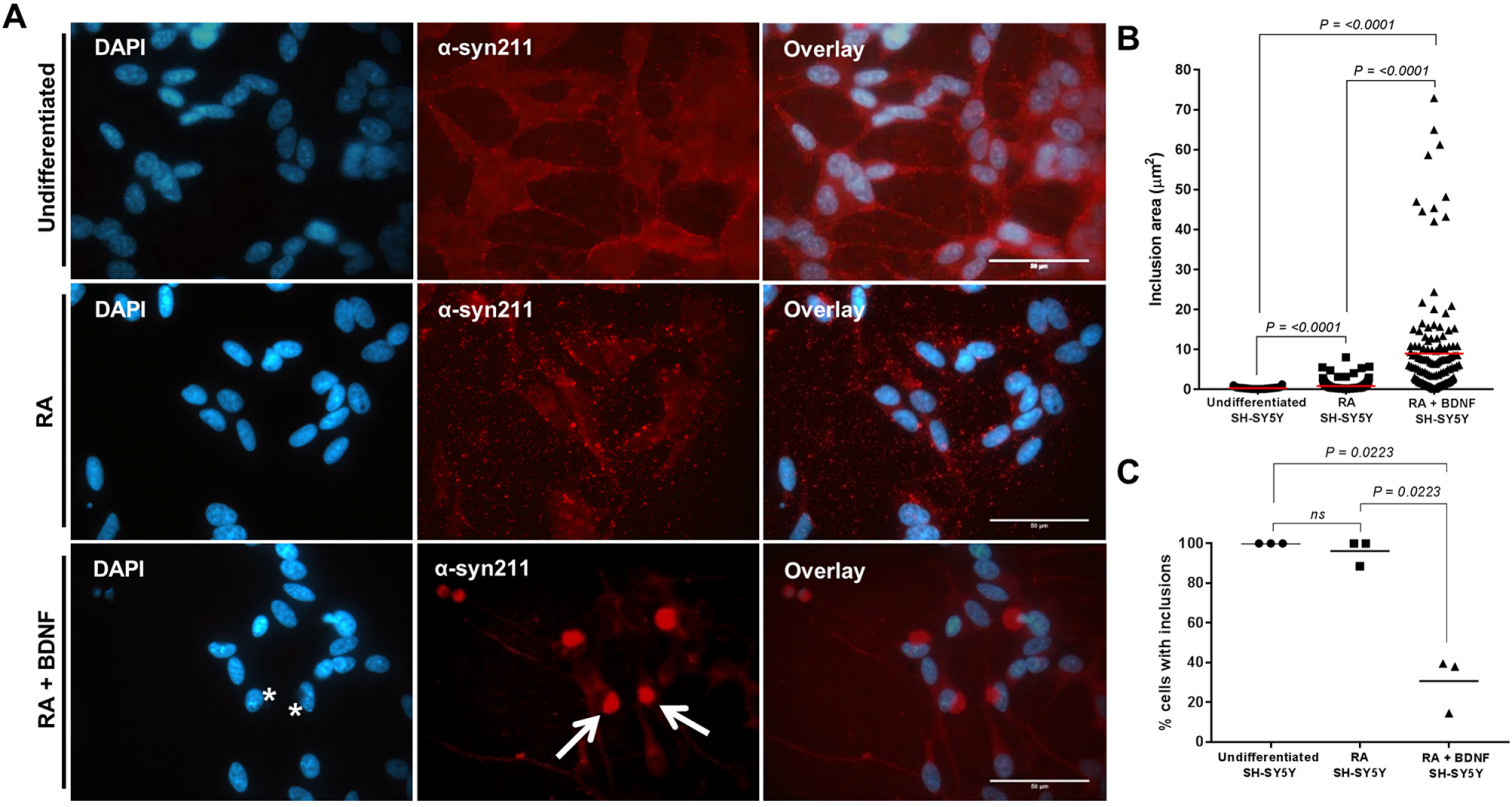
**Exogenous addition of α-syn oligomers induces the intracellular aggregation of α-syn in** **2D** **SH-SY5Y cultures.** (A) Double immunostaining of undifferentiated and differentiated SH-SY5Y cells reveals intracellular aggregation of α-syn following 24 h incubation with seeding oligomers. α-Syn inclusions in undifferentiated cells display several distinct, punctate accumulations dispersed throughout the cytoplasm, consistent with aggregates. In contrast, the RA+BDNF cell lines show a single prominent accumulation (red; arrows) and obscure the nucleus (asterisks). Scale bars: 50 µm. (B) Quantification of inclusion area (reflected as µm^2^) shows inclusions that are present in differentiated cells to be much larger than those in undifferentiated cells (measurements obtained from three independent experiments; *n=*50). All data points are present, with mean shown as a straight line. (C) The percentage of cells containing inclusions was determined by counting 100 cells per repeat. Cells were deemed to contain inclusions if aggregation matched that in reference images when compared to controls. Data points are presented from three independent biological repeats. (B,C) Differences between treatments were tested for significance using Kruskal–Wallis test with post-hoc Dwass-Steel-Chritchlow-Fligner for >6 data points and Conover-Inman for <6 data points. *P-*values are displayed in the figures.

Within the PD brain, LB pathology is only observed in a discrete cell population within *ex vivo* material (Braak et al., 2003). Here, we wanted to determine whether the extent of cellular differentiation resulted in alterations in the intracellular aggregation propensity of the cell line. Previous studies by us and others have shown that a distinct subtype of soluble oligomeric α-syn can seed intracellular aggregation of endogenous α-syn in undifferentiated SH-SY5Y lines (Danzer et al., 2009; Illes-Toth et al., 2015). Following addition of oligomeric α-syn to undifferentiated SH-SY5Y cells, the presence of multiple punctate α-syn-positive cytoplasmic inclusions with an average inclusion size of 0.28 µm^2^ was confirmed in our model (Fig. 2A,B). The addition of the same oligomer treatment on RA-differentiated cultures also produced several small cytoplasmic inclusions within each cell, but the average inclusion size was significantly larger (0.82 µm^2^) than in the undifferentiated SH-SY5Y cells (Fig. 2A,B). In both undifferentiated and RA-differentiated SH-SY5Y cells, all surviving cells developed the punctate inclusions (Fig. 2C).

In contrast, the addition of the same soluble oligomeric α-syn preparation to RA+BDNF-differentiated culture media demonstrated the development of a single perinuclear inclusion, consistent with a large LB-like aggregate (Fig. 2A, asterisks and arrows), with an average inclusion size of 8.95 µm^2^. Cellular inclusions could be detected 24 h after treatment, but only 31% of the cell population developed these structures (Fig. 2C). To our knowledge, this is the first observation of single large inclusions within a genetically unmodified cell culture model.

### SH-SY5Y differentiation in 3D culture retains a dopaminergic phenotype

Despite the promising initial phenotypic outcomes from the RA+BDNF-differentiated cells cultured in a 2D format, the protocol was not robust. These cells tended to detach from the culture vessel before the end of BDNF treatment and as such presented a wide variation in inclusion size (Fig. 2B) and viability (Fig. S2A). This detachment from the culture vessel meant that, although images could be produced of cells containing LB-like inclusions, the experiment itself was not repeatable enough for future investigations. We, therefore, sought to adapt our culturing procedure to allow for greater retention of cells after RA+BDNF differentiation. It was hoped that differentiation of these cells in a 3D matrix would stabilise the cell population during the development of LB-like inclusions and allow more robust handling methods during processing and analysis.

**Fig. 3.**
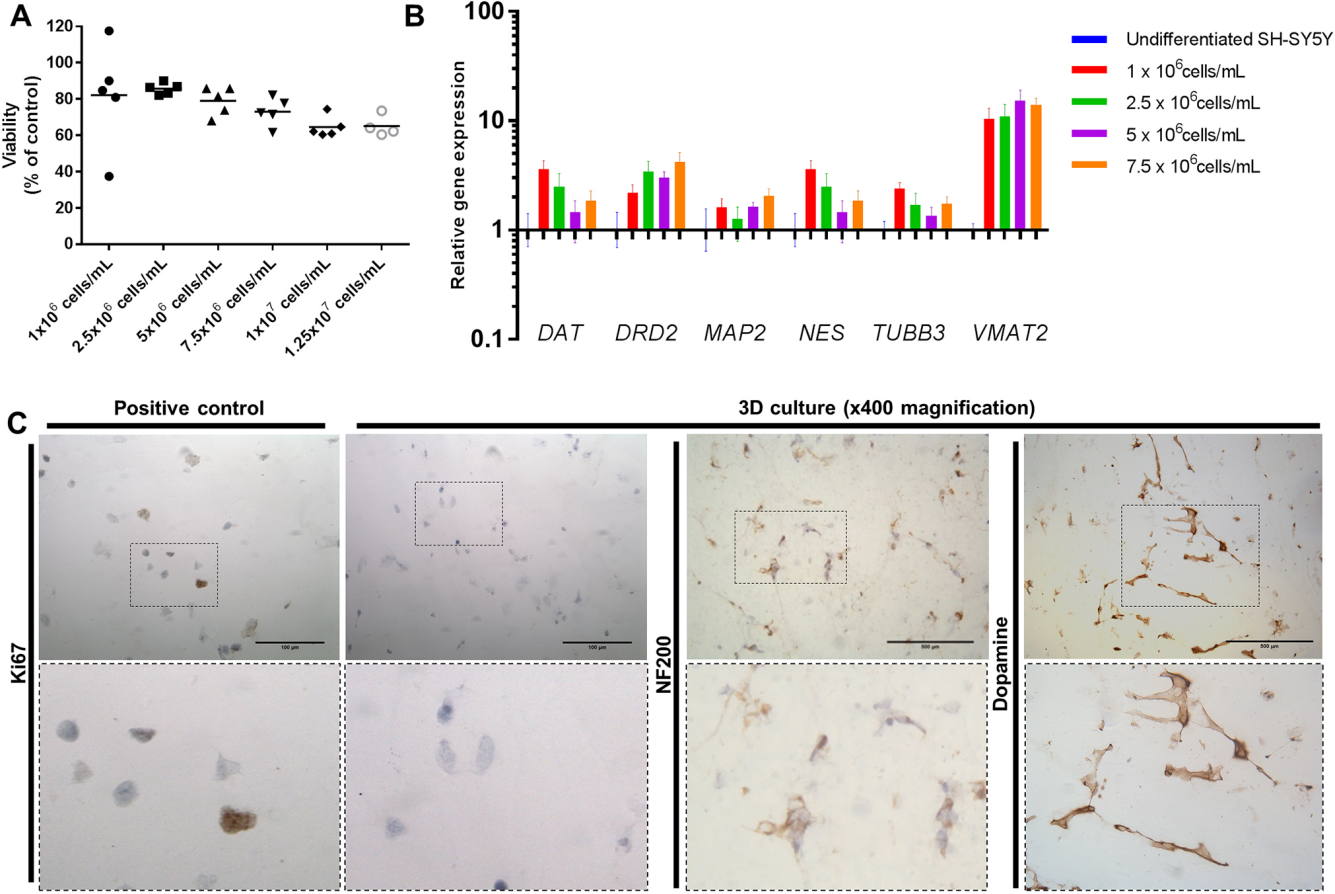
**Neuron-like cells cultured in 3D retain a dopaminergic phenotype.** (A) Cell number optimisation of 3D-differentiated SH-SY5Y using LDH cell viability assay. An incremental increase in cell density is associated with decreased cell viability (∼17% decrease from 1×10^6^ cells/ml to 1.25×10^7^ cells/ml). LDH cell viability assay was normalised to cell-density-matched positive controls. All data points are present, with mean shown as a straight line of at least four independent repeats. (B) mRNA levels of genes specifically associated with dopaminergic neurons were evaluated by qPCR in different cell densities. Relative gene expression normalised to reference genes (*ACTB* and *YWHAZ*) and undifferentiated SH-SY5Y. Expression of neuronal (*TUBB3*, *MAP2* and *NES*) and dopaminergic (*DAT*, *DRD2* and *VMAT2*) markers was evaluated in cell densities. A density of 7.5×10^6^ cells/ml demonstrated increased expression of *DRD2*, *MAP2* and *VMAT2*, and was chosen for further experiments. Data presented as mean±s.e.m. of at least three independent experiments. (C) 3D-differentiated SH-SY5Y cultures retain a post-mitotic neuronal dopaminergic phenotype in 3D as determined by the absence of Ki67 staining and positive staining for NF200 and dopamine. Positive control for Ki67 staining is undifferentiated SH-SY5Y cultured in 3D. Scale bars: 100 µm.

A variety of cell densities were used to evaluate the optimal cell conditions for differentiation in 3D. In 300 µl matrix volumes (in 24-well inserts), incremental increases in densities from 1×10^6^ cells/ml to 1.25×10^7^ cells/ml steadily decreased the viability of cells within the matrix (∼17%) as measured by lactate dehydrogenase (LDH) concentration (Fig. 3A). Upon differentiation in the 3D matrix, gene expression of *DRD2*, *MAP2* and *VMAT2* were increased (compared to undifferentiated SH-SY5Y) (Fig. 3B). A substantial increase in *VMAT2* expression within the same cells differentiated in 3D compared to 2D indicated the development of a more neuronal phenotype.

Based on these observations, a cell density of 7.5×10^6^ cells/ml was selected for further studies. At this cell density, the expression levels of *DAT*, *DRD2*, *MAP2*, *TUBB3* and *VMAT2* were all increased in relative abundance to the undifferentiated cells, and *NES* expression was decreased in comparison to other cell densities. Cells displayed a homogenous distribution within the matrix, in contrast to lower densities where clusters of cells were observed (Fig. S2B). In addition, this was the highest cell density at which cell viability was unaffected. Hence, 7.5×10^6^ cells/ml was the optimal balance between viability and homogeneity within the matrix. To demonstrate comparable differentiation of SH-SY5Y cultures after 7 days of treatment with RA+BDNF in 2D or 3D, all cells were confirmed to be immunonegative for Ki67 and immunopositive for NF200 and DA (Fig. 3C).

### Seeded inclusions in 3D cultures are reminiscent of human Lewy bodies

**Fig. 4.**
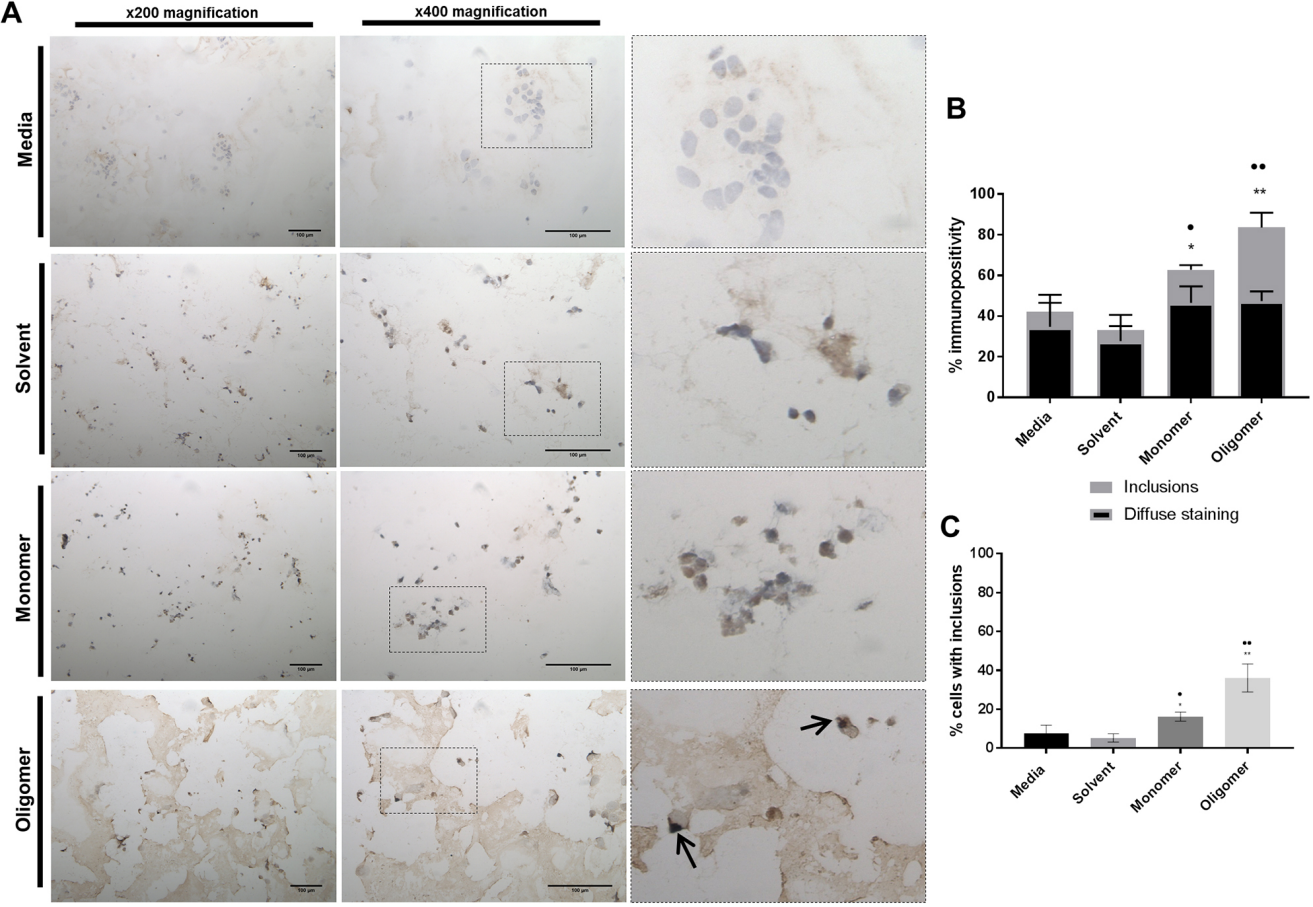
**Exogenous α-syn oligomers seed the development of intracellular inclusions in 3D SH-SY5Y cultures.** (A) Representative images of α-syn staining with syn211 monoclonal antibody (brown) with nuclei visualised using DAPI staining (blue/purple). α-syn oligomer treatment to cells leads to strong immunoreactivity for α-syn compared to isotype controls (Fig. S2), media only, or treatment with solvent or α-syn monomer. Arrows indicate the location of the intracellular inclusions. Scale bars: 100 µm. (B) Percentage of α-syn immunopositivity in 3D cultures treated for 24 h with media, solvent or α-syn in monomeric or oligomeric preparations. Cells were deemed to contain inclusions if aggregation matched that in reference images when compared to controls. Data presented as mean±s.e.m. of three independent experiments. (C) Monomeric and oligomeric α-syn treatments resulted in an increase in the percentage of cells with inclusions. Statistical significance in the oligomer-treated cultures related to the increase in large inclusions compared with media and solvent controls. Data presented as mean±s.e.m. of three independent experiments. (*, compared to media; •, compared to solvent). Evaluation of immunohistochemical staining was performed by counting 200 cells per slide, with ten slides counted per repeat (2000 cells counted in each treatment group for three independent experiments). (B,C) Statistical differences were determined using the Kruskal–Wallis test with *post hoc* Conover-Inman (*^/^^•^*P*≤0.05, **^/^^••^*P*≤0.01).

**Fig. 5.**
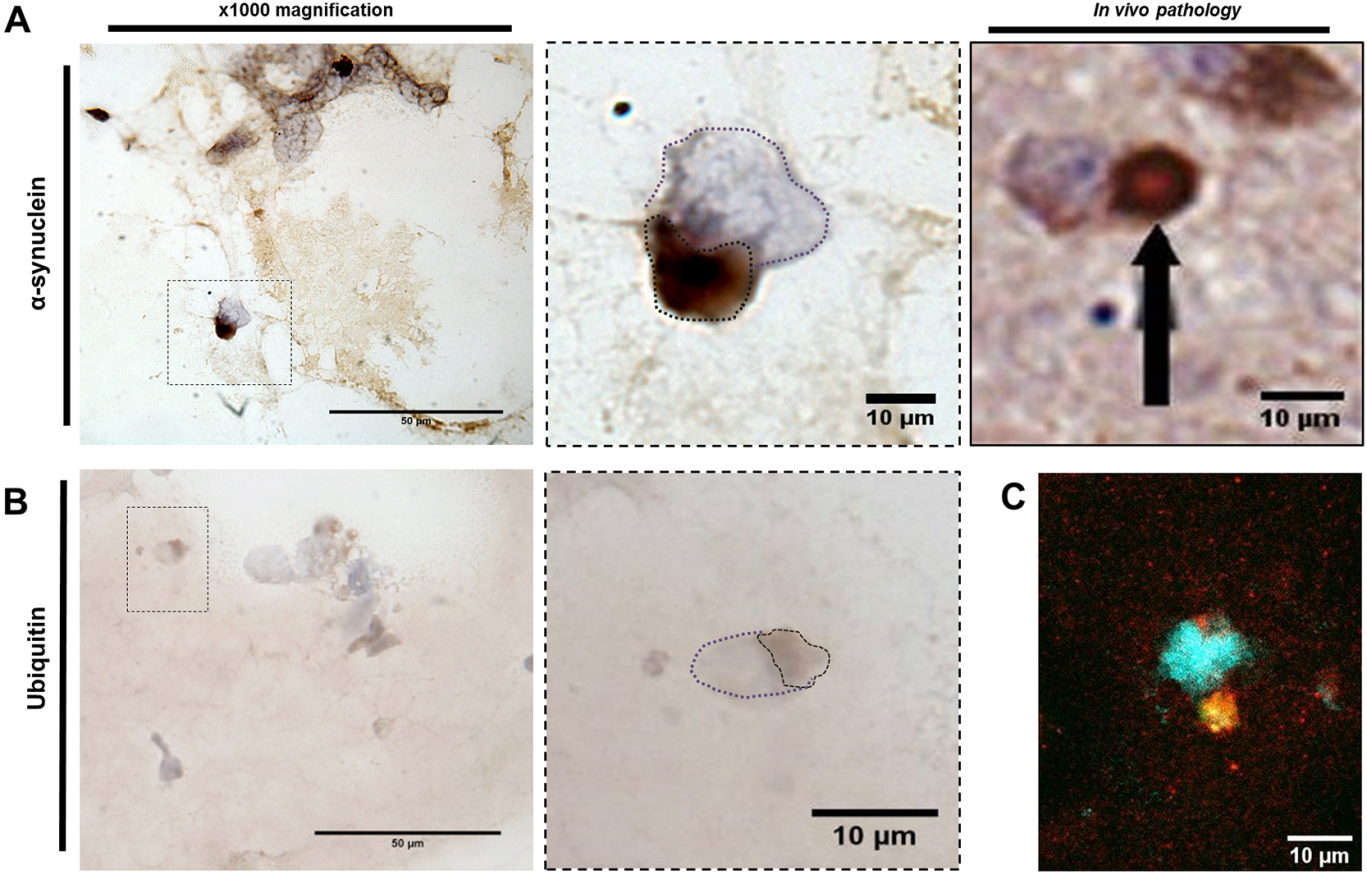
**α-Syn-seeded inclusions in 3D are indicative of *in vivo* Lewy body inclusions.** α-Syn oligomer treatments to 3D-differentiated cultures develop α-syn-positive inclusions that demonstrate the same morphology as *in vivo* Lewy bodies (LBs; A) and are positive for the marker ubiquitin (B). Dotted line in purple highlights cell nuclei, whereas brown dotted line highlights stained area. (C) Immunofluorescence shows colocalisation of α-syn (red) and ubiquitin (green) staining. Nuclei were visualised using DAPI staining (blue). Scale bars: 50 µm (left) and 10 µm (right). *In vivo* pathology image displayed with permission from Springer Nature. This image is not published under the terms of the CC-BY license of this article. For permission to reuse, please see Chesselet et al. (2012).

*In vivo* LBs are phenotypically more complex than the punctate aggregates of α-syn seen in 2D cell culture models. To determine the morphological changes in intracellular aggregates in 3D culture, fully differentiated cells within the matrix were treated with preformed α-syn oligomers. Similar to our observations in 2D cultures (Fig. 2), inclusions in our 3D cell culture model were detectable 24 h after α-syn oligomer treatment (Figs 4 and 5). Immunostaining of these nuclear-associated inclusions in our 3D model confirmed that they were α-syn positive (Fig. 4A) and an equivalent size to those seen *in vivo* (Fig. 5A). Fig. 4B shows the percentage of α-syn immunopositivity in 3D cultures treated for 24 h with media, solvent or α-syn in monomeric or oligomeric preparations. The data demonstrate that oligomer-treated cells develop significantly more inclusions than control cells. The percentage of cells containing observable inclusions also significantly increased relative to controls (Fig. 4C). Immunostaining for ubiquitin in the oligomer-treated 3D cell cultures demonstrated diffuse nuclear-associated staining that colocalised with the LB-like aggregates (Fig. 5B). Co-staining using both anti-α-syn and anti-ubiquitin antibodies demonstrates colocalisation of both proteins within the same inclusion (Fig. 5C). These inclusions are highly reminiscent of *in vivo* material (Shults, 2006), and completely absent in the isotype controls and untreated cultures (Fig. S3). Immunohistochemical detection of phosphorylated α-syn at S129 using a selective antibody was not able to show evidence of phosphorylation following 24 h treatment. This would indicate that the aggregates have either formed without the requirement for phosphorylation or that the S129 epitope is protected within these aggregates (Walker et al., 2013). The lack of observable phosphorylation within the inclusions would suggest that this post-translational modification may not be required for aggregate formation within this model, or occurs within a timeframe exceeding that used here. If so, then the LB-like inclusions observed here could represent an early aggregated morphology. Overall, the 3D differentiated SH-SY5Y culture model presented here can produce LB-like aggregates when treated with preformed oligomeric material that recapitulates characteristic *in vivo* LB.

## DISCUSSION

Previously, our group (Illes-Toth et al., 2015) and others (Danzer et al., 2007, 2009) have reported the induction of punctate intracellular aggregates in 2D undifferentiated cells when specific preparations of oligomeric α-syn are added to the media. Findings presented here describe a human 3D cell culture model that recapitulates LB-like formation. Inclusions are initiated by oligomeric α-syn preparations when introduced to terminally differentiated post-mitotic, neuron-like SH-SY5Y cells within a 3D matrix. Elevated expression of *TUBB3* and *MAP2* upon differentiation indicated stronger neuronal characteristics than when undifferentiated, and the detection of *DAT*, *DRD2* and *DA* confirmed a dopaminergic phenotype. Importantly, although the LB-like structures present in our model do not show evidence of phosphorylation, they are positive for α-syn and ubiquitin, closely resembling the morphology of inclusions found within the human pathology (Goedert et al., 2013).

Certain forms of α-syn located in the extracellular environment have recently been shown to be internalised by neurons through several receptor-mediated mechanisms and induce self-aggregation of endogenous α-syn (Bieri et al., 2018). The oligomers used in this study have previously been shown to induce the intracellular aggregation of α-syn by providing a nucleation site that recruits endogenous α-syn in the development of cell-associated aggregates (Danzer et al., 2009). This prion-like propagation has been proposed to explain the spread of the disease through the neuronal network and justify the post-mortem observations of α-syn aggregates in a PD patient that had undergone midbrain embryonic neuronal transplants (Braak et al., 2003; Kordower et al., 2008). Following internalisation, the ability of α-syn to trigger oligomerisation of endogenous α-syn potentially occurs through the oxidative or other stress responses (Deas et al., 2016; Di Maio et al., 2016). Although the precise series of events surrounding the development of these inclusions remains to be established, a model system such as the one described here opens the opportunity to investigate these mechanisms within a controlled environment and without the need to genetic modify or overexpress α-syn.

In multifactorial disorders such as PD, dissecting the complex pathological processes into simpler molecular events may allow us to better interpret disease progression. Using the 3D cell culture model described here may allow us to identify therapeutic interventions and the pathways leading to LB development before translating them to more complex models of the brain (e.g. transgenic mouse models) in which confounders such as neuroinflammation and innate immunity could mask the pathways required to be studied.

## MATERIALS AND METHODS

### Expression and purification of recombinant wild-type α-syn

Human WT α-syn was prepared as described previously (Smith et al., 2008). Briefly, α-syn was expressed in *Escherichia* *coli* BL21 (DE3) cells (New England Biolabs, Ipswich, MA, USA). Expression was induced using autoinduction media (Formedium, Norfolk, UK) at 37°C for 24 h. Bacterial cells were harvested by centrifugation at 4°C, 10,000 ***g*** and resuspended in lysis buffer [10 mM Tris-HCl (pH 8.0), 100 µg/ml lysozyme (Sigma-Aldrich, Poole, UK), 1 mM phenylmethylsulphonyl fluoride (PMSF; Sigma-Aldrich), 20 µg/ml DNase (Sigma-Aldrich), 20 µg/ml RNase (Sigma-Aldrich) and protease inhibitor cocktail (Life Technologies, Paisley, UK)]. The protein pellet was subjected to anion exchange followed by size exclusion chromatography. Lyophilised purified α-syn was stored at −20°C. Protein concentration was measured using a UV/vis spectrophotometer (CECIL 1000) at absorbance 280 nm with an extinction coefficient of 5960 M^−1^ cm^−1^.

### Preparation of seeding oligomers

Seeding oligomers were prepared as previously described by Danzer et al. (2009), omitting the addition of 10 µM FeCl_2_. Briefly, lyophilised α-syn was dissolved in 50 mM sodium phosphate buffer (pH 7.0) containing 20% ethanol to a final concentration of 7 µM. Following overnight incubation at 21°C with continuous shaking, oligomers were concentrated 1:14 using ultracentrifugation (VivaSpin 500 columns, MWCO 30 kDa, GE Healthcare, Buckinghamshire, UK), allowing the separation of oligomeric species from monomeric protein. Each batch of oligomers was used for experiments straight away.

### Cell culture, media and reagents

SH-SY5Y human neuroblastoma cells were purchased from the European Collection of Authenticated Cell Cultures (cat. no. 94030304; Public Health England, Salisbury, UK). All cell lines were confirmed to be mycoplasma free at 3-month intervals. Cell cultures were maintained at 37°C, 5% CO_2_ in DMEM high glucose with L-glutamine media (Lonza, Slough, UK) supplemented with 10% (v/v) foetal bovine serum (FBS; Life Technologies) and 1% (v/v) penicillin/streptomycin (P/S; Life Technologies); all experiments were completed within ten passages. For treatment with seeding oligomers, undifferentiated SH-SY5Y cells were allowed to adhere and expand to 70% confluence before treatment with monomeric α-syn, oligomeric α-syn or appropriate solvent controls for 24 h. For 2D differentiation into neuronal-like cells, cells were pre-differentiated in DMEM high glucose with L-glutamine supplemented with 10% (v/v) FBS, 1% (v/v) P/S and 10 µM all-trans-RA (Sigma-Aldrich). After 5 days in the presence of RA, cells were washed with serum-free DMEM and media changed to 50 ng/ml BDNF-supplemented (BDNF; cat. no. 450-02; PeproTech, London, UK) serum-free DMEM for 7 days. Following incubation, differentiated SH-SY5Y cells were treated with monomeric α-syn, oligomeric α-syn or appropriate solvent controls for 24 h at 10% v/v (0.01 mg/ml concentration referring to the monomer's concentration) (Illes-Toth et al., 2015; Danzer et al., 2007). Phase-contrast images of differentiated cells were captured using an Olympus Inverted microscope. Neurite length was measured using the free open source software, ImageJ 1.50i, whereby 200 neurites per treatment were measured, in three independent experiments.

### Immunofluorescence staining

For immunofluorescence staining, 2D cultures were fixed with ice-cold methanol for 15 min at −20°C followed by washing with phosphate buffered saline (PBS) solution containing 0.1% (v/v) Tween-20 (PBS-T; Sigma-Aldrich). After washing, unspecific binding sites were blocked with a solution containing PBS and 1% (w/v) bovine serum albumin (BSA; Sigma-Aldrich) for 1 h at room temperature under gentle continuous shaking. After blocking, cells were washed in PBS-T before incubation with primary antibody in blocking solution for 1 h at room temperature under gentle continuous shaking. The following antibody dilutions were used in this study: anti-α-synuclein (syn211; 1:2000 dilution; cat. no. 32-8100; Invitrogen, ThermoFisher Scientific, Loughborough, UK), anti-DA (1:200; ab6427; Abcam, Cambridge, UK), anti-hypophosphorylated NF200 (1:100; ab82259; Abcam) and anti-Ki67 (1:200; ab16667; Abcam). Following primary-antibody incubation, cells were washed with PBS-T and incubated with secondary antibody [Texas Red™ goat anti-mouse IgG (H+L); cat. no. 100125662; ThermoFisher Scientific] for 1 h at room temperature in the dark. Nuclei were counterstained with 0.1 µg/ml 4′,6-diamidino-2-phenylindole, dihydrochloride (DAPI; Sigma-Aldrich) for 5 min. Slides were mounted with immersion oil, sealed with nail varnish and left to cure overnight at 4°C in the dark prior to image capture using an Olympus BX60 brightfield/fluorescence microscope. Evaluation of immunofluorescence staining was performed by counting 200 cells per repeat, with immunopositive cells expressed as a percentage of total count. To quantify total fluorescence per cell, fluorescent images were analysed using the image analysis software ImageJ 1.50i using the CTCF calculation, in which the background fluorescence in a selected area is subtracted from the fluorescence value of a measured area, using the calculation: CTCF=[Integrated density−(Mean fluorescence reading of background×Fluorescence of area of selection)]/Cell number. Three independent biological repeats were performed in total.

### 3D cell culture and differentiation

Cells were pre-differentiated with RA in uncoated T75 flasks for 5 days prior to seeding into 3D Matrigel™ cultures. Matrigel™ was diluted to 6 mg/ml total protein with ice-cold serum-free DMEM and vortexed with cell pellets for 10 s, to a final cell concentration of approximately 7.5×10^6^ cells per ml of diluted Matrigel™. Using pre-chilled pipettes, Matrigel™/cell mixtures were transferred to either pre-warmed 8-well chamber slides or tissue culture inserts (ThinCerts; 0.4 µm pore size, Greiner Bio-One, Gloucestershire, UK), at 100 µl and 300 µl volumes, respectively, and incubated at 37°C for 1 h to form a colloidal gel. Following solidification of the 3D gels, pre-warmed serum-free DMEM supplemented with 50 ng/ml BDNF was added to the cultures and cells differentiated for a further 7 days. Media was changed every 3-4 days. After 7 days in 3D, cultures were treated with monomeric α-syn, oligomeric α-syn or appropriate solvent controls for 24 h at 10% v/v (0.01 mg/ml concentration referring to the monomer's concentration) (Illes-Toth et al., 2015; Danzer et al., 2007).

### Cryostat sectioning and histological staining of 3D cultures

For cryostat sectioning, 3D cultures grown in tissue inserts were frozen and stored at −80°C before sectioning. The frozen samples were cut into 10 µm sections (Leica CM3050 S Research Cryostat, Leica Microsystems, Newcastle upon Tyne, UK) at −25°C and mounted on X-tra^®^ adhesive glass slides (Leica Microsystems) and stored at −80°C prior to staining. For immunohistochemistry, 10 µm frozen sections from 3D thick-layer cultures were fixed with ice-cold 1:1 methanol and acetone, and endogenous peroxidases blocked. Staining with anti-α-synuclein monoclonal antibody (syn211; 1:100 dilution; Invitrogen) was performed using the Mouse on Mouse Polymer IHC Kit according to the manufacturer's protocols (ab127055; Abcam, Cambridge, UK). Staining with anti-ubiquitin (1:50 dilution; cat. no. ab7780; Abcam) rabbit primary antibodies was performed using the following method. Following Tris-buffered saline (TBS) washes, non-specific binding sites were blocked at room temperature for 2 h with 25% (v/v) normal goat serum (cat. no. 11819220; ThermoFisher Scientific, Loughborough, UK) and 1% (w/v) BSA in TBS. Sections were incubated with primary antibody overnight at 4°C. Slides were washed in TBS, and a 1:500 dilution of biotinylated goat anti-rabbit secondary antibody (cat. no. ab6720; Abcam) in 1% (w/v) BSA/TBS was applied for 30 min at room temperature. Binding of the secondary antibody was detected using a streptavidin-biotin complex (Vector Laboratories, Peterborough, UK) technique with 0.08% (v/v) hydrogen peroxide in 0.65 mg/ml 3,3-diaminobenzidine tetrahydrochloride (DAB; Sigma-Aldrich) in TBS. Sections were counterstained with Mayer's Haematoxylin (Leica Microsystems), dehydrated, cleared and mounted with Pertex mounting medium (Leica Microsystems). Evaluation of immunohistochemical staining was performed by counting 200 cells per slide, with ten slides counted per repeat (2000 cells counted in each treatment group for three independent experiments); immunopositive cell values are expressed as a percentage of the total count.

### Immunofluorescence of 3D cultures

For immunofluorescence of 3D cultures, frozen sections were fixed with 1:1 ice-cold methanol:acetone. Blocking was performed using the Mouse on Mouse Polymer IHC Kit according to the manufacturer's protocols (ab127055; Abcam). Slides were incubated with anti-α-syn (syn211; 1:100 dilution; Invitrogen) and anti-ubiquitin (1:1000; cat. no. ab7780; Abcam) primary antibodies for 1 h at room temperature. Following PBS-T washes, secondary antibodies [Alexa Fluor^®^ 488 goat anti-rabbit IgG (H+L) cat. no. ab150077, Abcam, and Texas Red™ goat anti-mouse IgG (H+L) cat. no. 100125662, ThermoFisher Scientific] were incubated at 1:1000 dilution for 1 h at room temperature. Nuclei were counterstained with 0.1 µg/ml DAPI (Sigma-Aldrich) for 5 min. Slides were mounted with immersion oil, sealed with nail varnish and left to cure overnight at 4°C in the dark before image capture using a Leica LSM 800 confocal microscope.

### RNA extraction, cDNA synthesis and qPCR

Total RNA was extracted using either TRIzol^®^ Reagent (Life Technologies) or the ReliaPrep™ RNA Cell Miniprep System (Promega, Southampton, UK) according to the manufacturers’ protocols. Complementary DNAs (cDNAs) were synthesised by the nanoScript 2 Reverse Transcriptase kit (Primerdesign, Chandler's Ford, UK) from equal amounts of purified RNA (1 µg). Target genes were investigated using qPCR conducted on either undifferentiated or differentiated SH-SY5Y populations using pre-validated primer sets (Primerdesign). Reaction volumes of 20 µl were prepared using PrecisionPLUS 2× Real-Time PCR MasterMix premixed with ROX and SYBR green (Primerdesign). The amplification was performed under the following conditions: 2 min at 95°C and then 40 cycles at 95°C for 15 s and 60°C for 60 s followed by a post-PCR melt-curve analysis performed on a StepOnePlus™ Real-Time PCR System (Applied Biosystems, Lutterworth, UK). The sizes of PCR products were confirmed using agarose gel electrophoresis. Reference gene analysis was undertaken using the geNormPLUS kit (Primerdesign) and Qbase+ software (Primerdesign) to identify inherently stable reference genes between different treatment groups. Gene expression levels were normalised against reference genes *ACTB* (β-actin) and *YWHAZ* (tyrosine 3-monooxygenase/tryptophan 5-monooxygenase activation protein zeta polypeptide) for each sample and fold changes were calculated using the 2^−ΔΔCt^ method (Schmittgen and Livak, 2008) by setting the expression levels of each gene in undifferentiated SH-SY5Y cells as 1. Data collected from at least three independent biological replicates was calculated and plotted as mean log_10_-fold changes ±s.e.m.

### Cell viability

Cell viability of 2D or 3D cultures was analysed with either resazurin (mitochondrial activity) or lactate dehydrogenase (LDH; cell membrane integrity) levels. For the resazurin reduction assay, a 3 mg/ml stock solution of resazurin was prepared in cell culture media and further diluted to 10 µg/ml. Following treatment with oligomeric or monomeric species and appropriate solvent controls, media was removed and 100 µl of culture media with 10 µg/ml resazurin was incubated with the cells for 2 h. Fluorescence (Ex_530nm_/Em_590nm_) was measured on a CLARIOstar^®^ plate reader (BMG LABTECH, Buckinghamshire, UK). Culture media was incubated with resazurin in parallel as a blank control. As a positive control, cells were incubated with 1% (v/v) Triton X-100. Cell viability was calculated as a percentage of live cells normalised to negative controls minus relative fluorescence intensity (RFU) of blank wells. For LDH assay, the Pierce LDH Cytotoxicity Assay Kit was used according to the manufacturer's instructions (cat. no. 88953; ThermoFisher Scientific). Briefly, 50 µl of medium from each well was transferred to a 96-well plate, followed by addition of 50 µl of reaction mixture, and incubated for 30 min at room temperature in the dark. Cell culture media was included in parallel as a blank control. After addition of 50 µl stop solution, absorbance measurements at 490 nm and 680 nm were recorded on a CLARIOstar^®^ plate reader (BMG LABTECH). The *A*_680nm_ (background signal from the instrument) was subtracted from the *A*_490nm_ followed by subtraction of blanks. Percentages of live cells were determined by normalisation of the absorbance values from the test sample to positive controls.

### Statistical analysis

Statistical analysis was undertaken using StatsDirect version 3.0.126. Data was unpaired and determined to be non-parametric following the Shapiro–Wilk test of normality; therefore, a Kruskal–Wallis test was undertaken with *post hoc* analysis. *Post hoc* analysis was only undertaken if the initial significance test was at *P*<0.05. Dwass-Steel-Chritchlow-Fligner was used for data points >6 and Conover-Inman *post hoc* analysis for <6 data points. *P-*values are denoted on the graphs. Results of all statistical analysis performed are shown in Table S1.

## Supplementary Material

Supplementary information
