# Supplementary material for: Recapitulating Parkinson's disease pathology in a three-dimensional human neural cell culture model
Source: Dis Model Mech. 2019 Apr 9;12(4):dmm038042. doi: 10.1242/dmm.038042 (PMC6505482; doi:10.1242/dmm.038042)
Supplement: Supplementary information [file dmm-12-038042-s1.pdf]

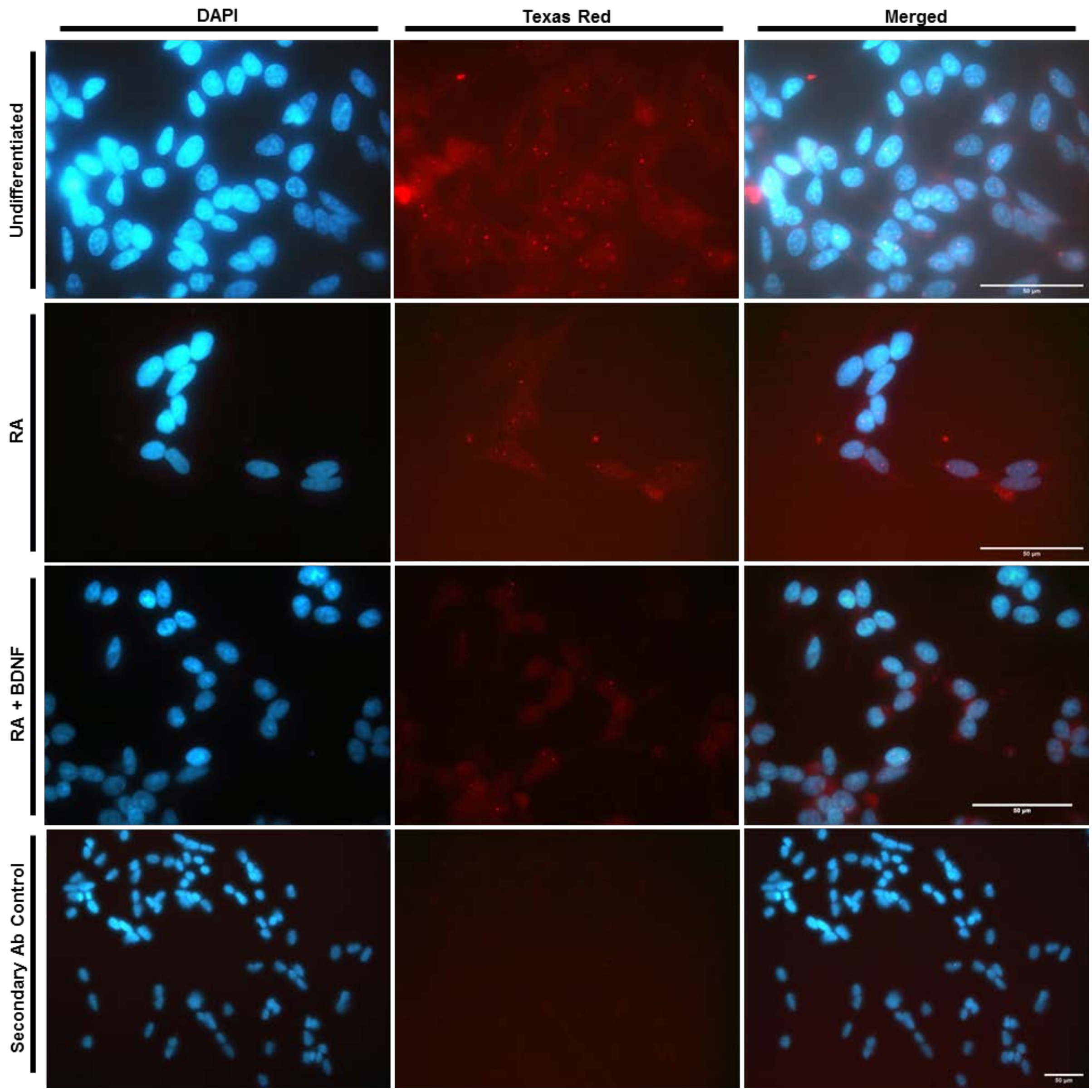

Figure S1. Double-immunostaining of undifferentiated, RA ad RA+BDNF SH-SY5Y with tyrosine hydroxylase (*red*) reveals expression of the protein in all treatment groups. Undifferentiated SH-SY5Y were stained as a secondary antibody control which revealed absence of unspecific staining. Nuclei were visualised using DAPI staining (*blue*). Scale bars represent 50 µm.

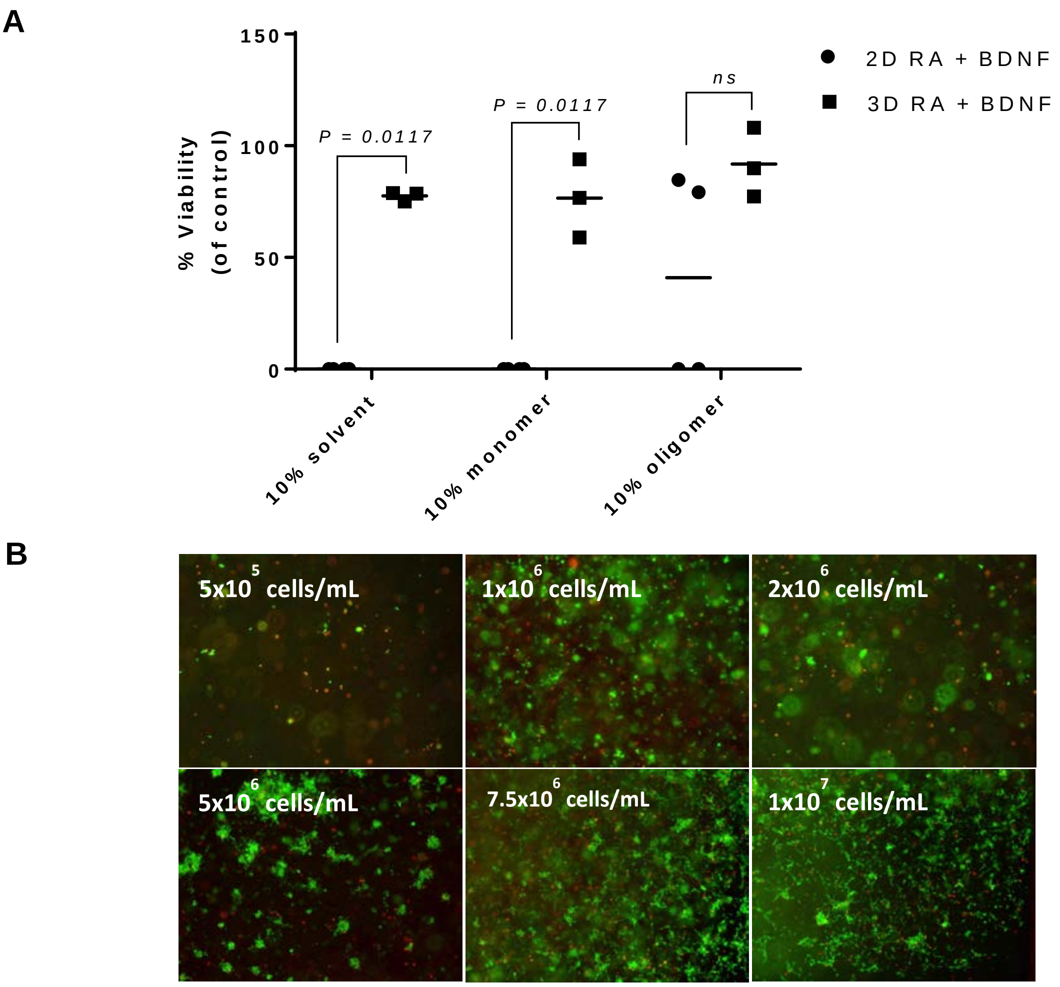

Figure S2. A. Alamar blue cell viability assay of 2D and 3D cultures exposed to monomeric and oligomeric α-syn and solvent controls. Treatment with seeding oligomers doesn't induce a significant amount of cell death in undifferentiated and RA-differentiated cultures. RA +BDNF SH-SY5Y are sensitive to solvent treatment, related to the cells state following differentiation, which is rescued by culturing RA-BDNF cell in a 3D format. All data points are present from at least three independent experiments, with mean shown as a straight line. Data presented as mean and SEM of at least 3 independent experiments. **B.** LIVE (*green*)/ DEAD (*red*) staining reveals the distribution of cells throughout the 3D matrix.

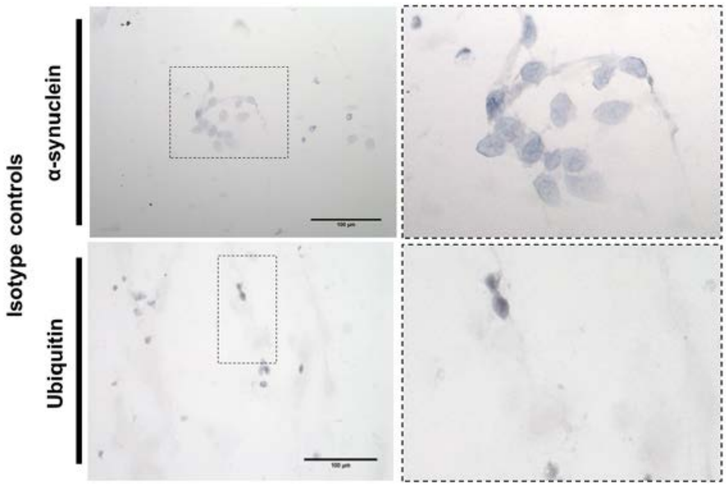

Figure S3. Immunohistochemistry isotype controls for α-syn and ubiquitin staining reveals the absence of non-specific background signal.

Disease Models & Mechanisms • Supplementary information

**Figure number**	**Results of initial statistical test (Kruskal Wallis)**	**Results of post-hoc analysis**					
**Figure 1A**	*P* = <0.0001	Undiff vs. RA **P = <0.0001**	Undiff vs. RA+BDNF **P = <0.0001**	RA vs. RA+BDNF **P= <0.0001**			
**Figure 1B**	*P* = 0.0001	Undiff vs. RA **P = 0.0022**	Undiff vs. RA+BDNF *P* = 0.1046	RA vs. RA+BDNF **P = 0.0015**			
**Figure 1C**	*P* = <0.0001	Undiff vs. RA **P = 0.0002**	Undiff vs. RA+BDNF **P = <0.0001**	RA vs. RA+BDNF *P* = <0.999			
**Figure 1 D**	*P* = 0.0001	Undiff vs. RA **P = 0.0012**	Undiff vs. RA+BDNF *P* = 0.919	RA vs. RA+BDNF **= 0.0005**			
**Figure 1 E-DRD2**	*P* = 0.0321	Undiff vs. RA *P* = 0.5024	Undiff vs. RA+BDNF **P = 0.0114**	RA vs. RA+BDNF **P = 0.0043**			
**Figure 1 E-DAT**	ns at: *P* = 0.7217	No further statistical analysis					
**Figure 1 E-NES**	ns at: *P* = 0.1887	No further statistical analysis					
**Figure 1 E-MAP2**	ns at: *P* = 0.0575	No further statistical analysis					
**Figure 1 E-VMAT**	ns at: *P* = 0.1784	No further statistical analysis					
**Figure 1 E-TUBB3**	ns at: *P* = 0.5798	No further statistical analysis					
**Figure 2B**	*P*= <0.0001	Undiff vs. RA **P = <0.0001**	Undiff vs. RA+BDNF **P = <0.0001**	RA vs. RA+BDNF **P = <0.0001**			
**Figure 2C**	*P*= 0.0221	Undiff vs. RA *P* = >0.9999	Undiff vs. RA+BDNF **P = 0.0223**	RA vs. RA+BDNF **P = 0.0223**			
**Figure 1 E-DRD2**	ns at: *P* = 0.1413	No further statistical analysis					
**Figure 1 E-DAT**	ns at: *P* = 0.7125	No further statistical analysis					
**Figure 1 E-NES**	ns at: *P* = 0.3107	No further statistical analysis					
**Figure 1 E-MAP2**	ns at: *P* = 0.2477	No further statistical analysis					
**Figure 1 E-VMAT**	ns at: P = 0.0696	No further statistical analysis					
**Figure 1 E-TUBB3**	ns at: *P* = 0.0544	No further statistical analysis					
**Figure 4B**	*P*= 0.0329	Media vs. Solvent *P* = 0.6811	Media vs. Monomer **P = 0.047**	Media vs. Oligomer **P = 0.0021**	Solvent vs. Monomer **P = 0.0242**	Solvent vs. Oligomer **P = 0.0012**	Monomer vs. Oligomer *P* = 0.0656
**Figure 4C**	*P* = 0.0329	Media vs. Solvent *P* = 0.6811	Media vs. Monomer **P = 0.047**	Media vs. Oligomer **P = 0.0021**	Solvent vs. Monomer **P = 0.0242**	Solvent vs. Oligomer **P = 0.0012**	Monomer vs. Oligomer *P* = 0.0656
**Supplementary Figure 2A**	Solvent- *P* = 0.0193	2D vs. 3D *P* = 0.0117					
**Supplementary Figure 2A**	Monomer- *P* = 0.0193	2D vs. 3D *P* = 0.0117					
**Supplementary Figure 2A**	Oligomer- *P* = 0.1536	No further statistical analysis					

Table S1. Results of all statistical analysis undertaken.
